# Impact of data synthesis strategies for the classification of craniosynostosis

**DOI:** 10.3389/fmedt.2023.1254690

**Published:** 2023-12-13

**Authors:** Matthias Schaufelberger, Reinald Peter Kühle, Andreas Wachter, Frederic Weichel, Niclas Hagen, Friedemann Ringwald, Urs Eisenmann, Jürgen Hoffmann, Michael Engel, Christian Freudlsperger, Werner Nahm

**Affiliations:** ^1^ Institute of Biomedical Engineering (IBT), Karlsruhe Institute of Technology (KIT), Karlsruhe, Germany; ^2^ Department of Oral, Dental and Maxillofacial Diseases, Heidelberg University Hospital, Heidelberg, Germany; ^3^ Institute of Medical Informatics, Heidelberg University Hospital, Heidelberg, Germany

**Keywords:** statistical shape model, generative adversarial network, GAN, craniosynostosis, classification, CNN, photogrammetric surface scan, PCA

## Abstract

**Introduction:**

Photogrammetric surface scans provide a radiation-free option to assess and classify craniosynostosis. Due to the low prevalence of craniosynostosis and high patient restrictions, clinical data are rare. Synthetic data could support or even replace clinical data for the classification of craniosynostosis, but this has never been studied systematically.

**Methods:**

We tested the combinations of three different synthetic data sources: a statistical shape model (SSM), a generative adversarial network (GAN), and image-based principal component analysis for a convolutional neural network (CNN)–based classification of craniosynostosis. The CNN is trained only on synthetic data but is validated and tested on clinical data.

**Results:**

The combination of an SSM and a GAN achieved an accuracy of 0.960 and an F1 score of 0.928 on the unseen test set. The difference to training on clinical data was smaller than 0.01. Including a second image modality improved classification performance for all data sources.

**Conclusions:**

Without a single clinical training sample, a CNN was able to classify head deformities with similar accuracy as if it was trained on clinical data. Using multiple data sources was key for a good classification based on synthetic data alone. Synthetic data might play an important future role in the assessment of craniosynostosis.

## 1. Introduction

Craniosynostosis is a group of head deformities affecting infants involving the irregular closure of one or multiple head sutures and its prevalence is estimated to be between four and 10 cases per 10,000 live births (1). As described by Virchow’s law (2), depending on the affected suture, distinct types of head deformities arise. Genetic mutations have been identified as one of the main causes of craniosynostosis (3, 4), which has been linked to increased intracranial pressure (5) and decreased brain development (6). The most-performed therapy is surgical intervention consisting of resection of the suture and cranial remodeling of the skull. It has a high success rate (7) and is usually performed within the first 2 years of age. Early diagnosis is crucial and often involves palpation, cephalometric measurements, and medical imaging. Computed tomography (CT) imaging is the gold standard for diagnosis, but it makes use of harmful ionizing radiation which should be avoided, especially for very young infants. Black-bone magnetic resonance imaging (MRI) (8) is sometimes performed, but requires sedation of the infants to impede moving artifacts. 3D photogrammetric scanning enables the creation of 3D surface models of the child’s head and face and is a radiation-free, cost-effective, and fast option to quantify the head shape. It can be employed in a pediatrician’s office and has potential to be used with smartphone-based scanning approaches (9).

Due to its low prevalence, craniosynostosis is included in the list of rare diseases by the American National Organization for Rare Disorders. Due to limited data, strict patient data regulations, and difficulties in anonymization (photogrammetric recordings show head and face), there are no publicly available clinical datasets of craniosynostosis patients available online. Synthetic data based on clinical data could potentially be used as a substitute to develop algorithms and approaches for the assessment of craniosynostosis, but so far only one synthetic dataset based on a statistical shape model (SSM) from our group (10) has been made publicly available. Scarce training data and high class imbalance due to the different prevalences of the different types of craniosynostosis (4) call for the usage of synthetic data to support or even replace clinical datasets as the primary resource for deep learning (DL)–based assessment and classification. The inclusion of synthetic data could facilitate training due to the reduction of class imbalance and increase the classifier’s robustness and performance. In addition, synthetic data may also be used as a cost-effective way to acquire the required training material for classification models without manually labeling and exporting a lot of clinical data. Using synthetic data for classification studies in a supporting manner or as a full replacement for clinical data has gained attraction in several fields of biomedical engineering (11, 12), especially if clinical data are not abundant. While the classification approaches of craniosynostosis on computed tomography (CT) data (13), 2D images (14), and 3D photogrammetric surface scans (15–17) have been proposed, the dataset sizes were below 500 samples [e.g. (17, 15, 13)] and contained high class imbalances. The usage of synthetic data is a straightforward way to increase training size and stratify class distribution.

However, although the need for synthetic data had been acknowledged (15), synthetic data generation for the classification of head deformities has not been systematically explored yet. With the scarce availability of clinical data and multiple options of synthetic data generation available, we aim to test the effectiveness of multiple data synthesis methods both individually and as multi-modal approaches for the classification of craniosynostosis. Using synthetic data as training material facilitates not only the development of larger and more robust classification approaches but also makes data sharing easier and increases data availability. A popular approach for 3D data synthesis is statistical shape modeling. It describes the approach to model 3D geometric shape variations by means of statistical analysis. With the application of head deformities, they have been employed to distinguish clinical head parameters (18), to evaluate head shape variations (19), to assess therapy outcome (20), and to classify craniosynostosis (16). Although their value in the clinical assessment of craniosynostosis has been shown, the impact of SSM-based data augmentation for the classification of craniosynostosis has not been evaluated yet. With the introduction of a conversion of the 3D head geometry into a 2D image, image-based convolutional neural network (CNN)–based classification (17) can be applied on low-resolution images. Generative adversarial networks (GANs) (21) have been suggested as a data augmentation tool (15) and have been able to increase classification performance for small datasets (22).

The goal of this work is to employ a classifier based on synthetic data, using three different types of data synthesis strategies, which can create any number of samples based on a set of clinical training data: SSM, GAN, and image-based principal component analysis (PCA). The three modalities are systematically compared regarding their capability in the classification of craniosynostosis when trained only on synthetic data. We will demonstrate that the classification of craniosynostosis is possible with a multi-modal synthetic dataset with a similar performance to a classifier trained on clinical data. In addition, we propose a GAN design tailored toward the creation of low-resolution images for the classification of craniosynostosis. The GAN, the different SSMs, and PCA, were made publicly available along as all the 2D images from the synthetic training, validation, and test sets.

## 2. Methods

### 2.1. Dataset and preprocessing

All data from this study were provided from the Department of Oral and Maxillofacial Surgery of the Heidelberg University Hospital, in which patients with craniosynostosis are routinely recorded for therapy planning and documentation purposes. The recording device is a photogrammetric 3D scanner (Canfield VECTRA-360-nine-pod system, Canfield Science, Fairfield, NJ, USA). We used a standardized protocol that had been examined and approved by the Ethics Committee Medical Faculty of the University of Heidelberg (Ethics number S-237/2009). The study was carried out according to the Declaration of Helsinki, and written informed consent was obtained from parents.

**Figure 1:**
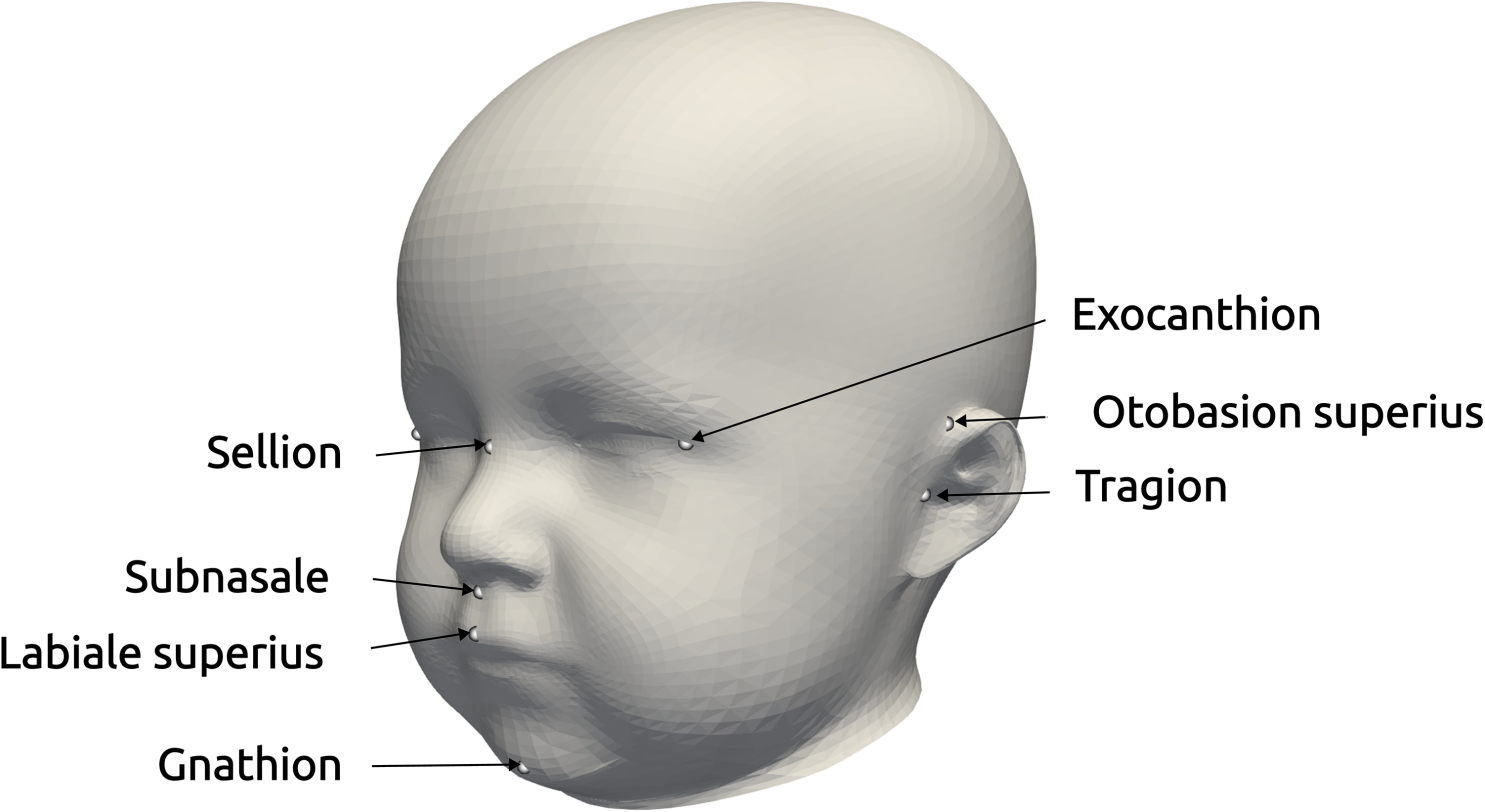
Landmarks provided in the dataset, used for the alignment for statistical shape modeling and the coordinate system creation of the distance maps (17). The three landmarks on the right exist for both left and right parts of the head.

**Figure 2:**
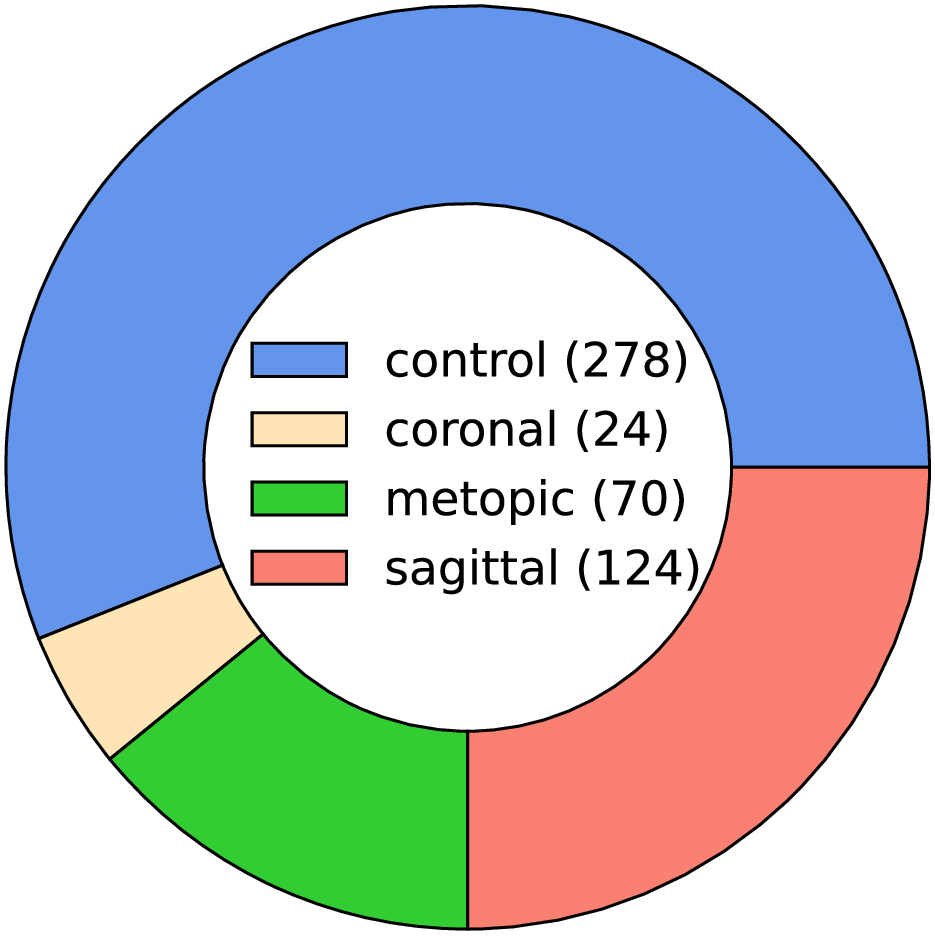
Pie chart of the class ratios in the clinical dataset (control 56%, coronal 5%, metopic 14%, and sagittal 25%). The legend in the center shows the absolute number of samples in the dataset (496 samples in total).

**Figure 3:**
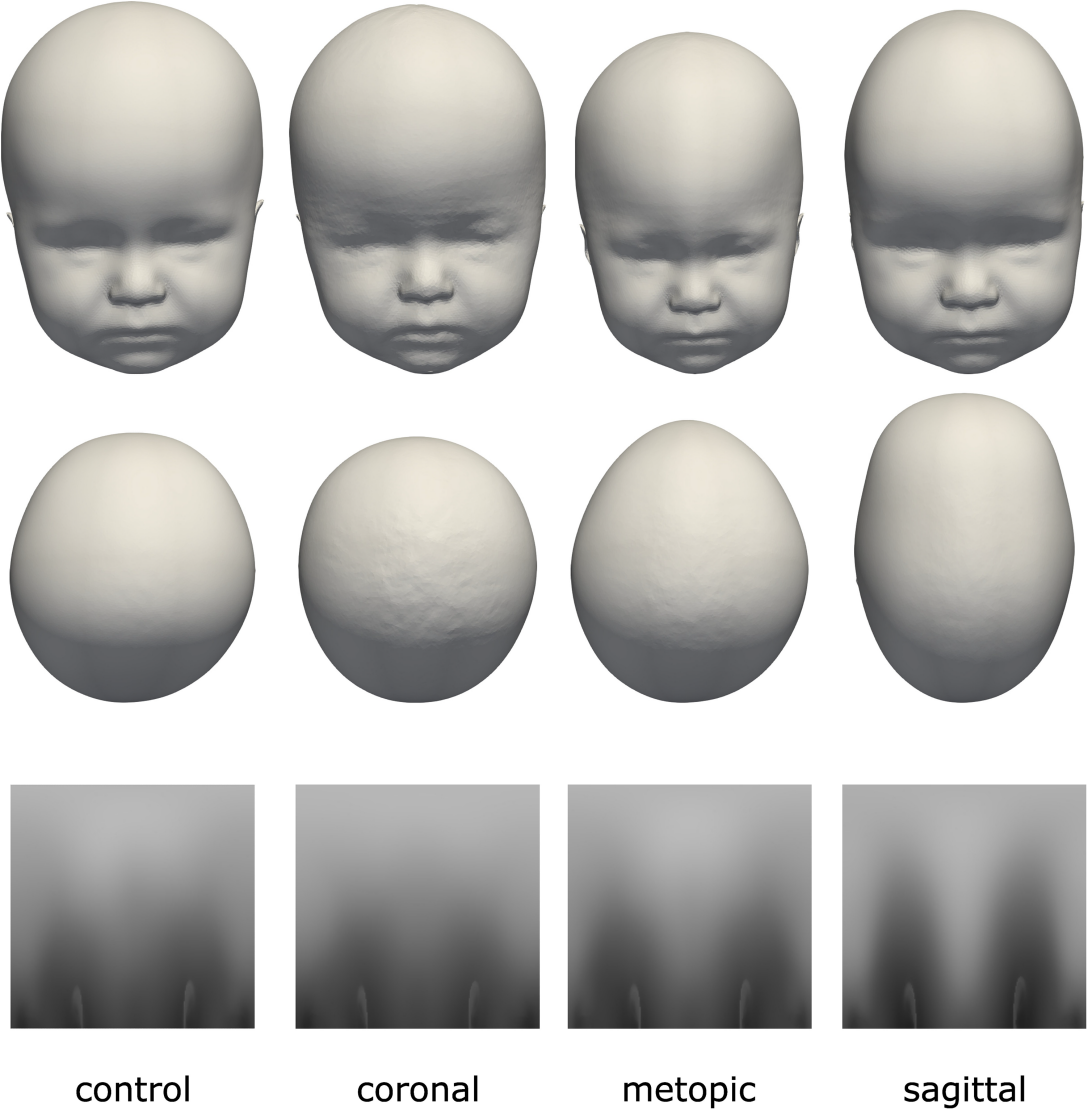
The four classes of the dataset with their distinct head shapes and their resulting distance maps representation. Top row: frontal view; middle row: top view; bottom row: 2D distance maps.

Each data sample was available as a 3D triangular surface mesh. We selected the 3D photogrammetric surface scans from all available years (2011–2021). If multiple scans for the same patient were available, we selected only the latest preoperative scan to avoid duplicate samples of the same patients. All patient scans had been annotated by medical staff with their diagnosis and 10 cephalometric landmarks. Figure 1 shows the available landmarks on the dataset. We retrieved patients with coronal suture fusion (brachycephaly and unilateral anterior plagiocephaly), sagittal suture fusion (scaphocephaly), and metopic suture fusion (trigonocephaly), as well as a control group with the dataset distribution displayed in Figure 2. Besides healthy subjects, the control group also contained patients suffering from mild positional plagiocephaly without suture fusion. Subjects with positional plagiocephaly in the control group were treated with helmet therapy or laying repositioning. In contrast, all patients suffering from craniosynostosis required surgical treatment and underwent remodeling of the neurocranium. The four head shapes resulting from craniosynostosis are visualized in Figure 3.

We used the open-source Python module pymeshlab (23) (version 2022.2) to automatically remove some recording artifacts such as duplicated vertices and isolated parts. We also closed holes resulting from incorrect scanning and removed irregular edge lengths by using isotropic explicit re-meshing (24) with a target edge length of 1 mm. In an earlier work (17), we defined a 2D encoding of the 3D head shape (“distance maps,” displayed in Figure 3, bottom row), which was also included in the pre-processing pipeline with the default parameters of (17).

### 2.2. Data subdivision

We aimed to test multiple data generation models (GAN, SSM, and PCA) to create training material for the classification. It was therefore required to strictly separate the data from which we trained the generative models to the data on which we evaluated the classification performance. The test set had to be strictly unknown to the classification model to avoid leakage (an overestimation of the model performance due to statistical information from the training or validation set “leaking” into the test set).

**Figure 4:**
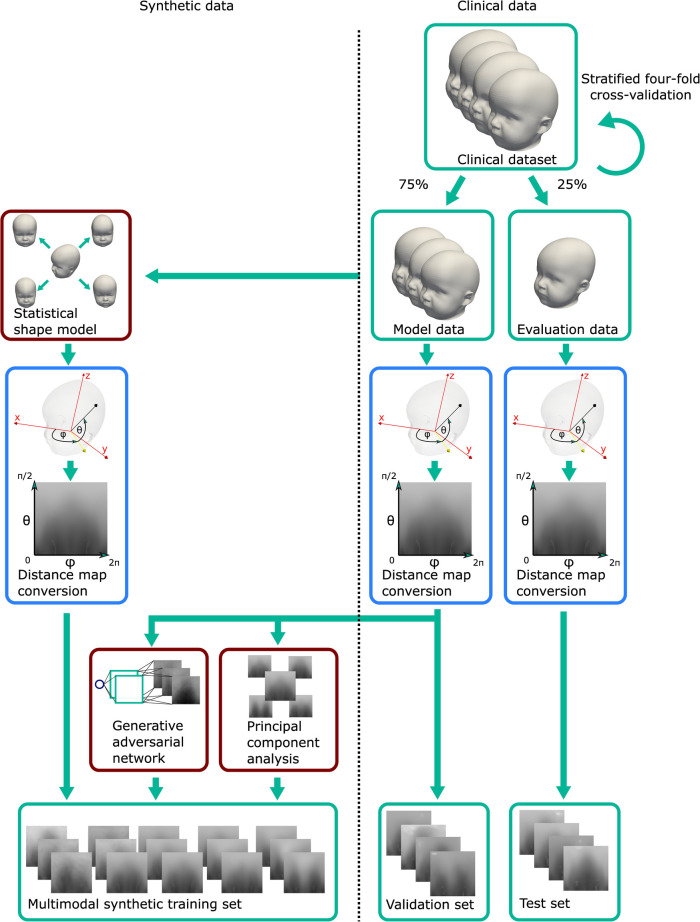
Data subdivision for the creation of synthetic data and the datasets for the classification experiment. The “model data” were used to produce the synthetic samples on which the CNN was trained and for creating the validation set. The “evaluation data” comprised the other part of the clinical data and were used to create the 2D images for the test set for the CNN. Green: data; blue: 3D–2D image conversion; dark red: generative models.

We introduce a terminology for this study design, which is also visually depicted in Figure 4. The clinical dataset is split in a stratified fourfold cross-validation scenario into 75% “model data” and 25% “evaluation data” (the stratification refers to the class distribution, so the same class ratio as shown in Figure 2 was present in both splits).

The “model data” was clinical data which had two purposes: Its first purpose was to serve as training data for the three generative models. Those generator models would in turn synthesize the training sets for the CNN classification model. Second, converted into the 2D domain, the “model data” served as the validation set for the CNN classification model. The “evaluation data” was composed of the remaining 25% of the clinical data and was strictly separated from the other data only to be used in the 2D distance maps domain as the test set for the CNN classification model. This set was therefore a true independent test set since it was never seen either during creation of the data synthesizers or during training of the CNN classification.

As the CNN classification model operated on 2D images, all 2D images were created from each 3D surface scan as 28×28-sized craniosynostosis distance maps, which was sufficient for good classification in an earlier study (17). Each of the synthetic data generators, SSM, GAN, and PCA, are described below.

### 2.3. Data synthesis

#### 2.3.1. Statistical shape model

**Figure 5:**
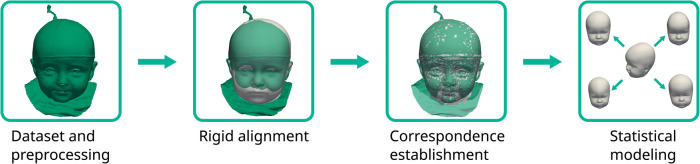
The statistical shape model pipeline employed in this study. The target scan is colored green with the deforming template in white.

The pipeline for the SSM creation was similar to Dai et al. (25) and consisted of initial alignment, dense correspondence establishment, and statistical modeling to extract the mean shape and the principal components from the sample covariance matrix (see also Figure 5). For correspondence establishment, we employed template morphing.

We used the mean shape of our previously published SSM (10) as a template, which would be morphed onto each of the target scans. Procrustes analysis was employed on the 10 cephalometric landmarks to obtain a transformation including translation, rotation, and isotropic scaling from the template to each target according to the cephalometric landmarks on the face and ears. For correspondence establishment, we employed the Laplace–Beltrami regularized projection (LBRP) approach (26) to morph the template onto each of the targets. We used two iterations: a high stiffness fit (providing a now landmark-free transformation from template to the target, improving the alignment also from the back of the head not covered with the landmarks) and a low stiffness fit (allowing the template to deform very close to the targets (27)). The deformed templates were then in dense correspondence, sharing the same point IDs across all scans and were used for further processing.

GPA was performed to remove both rotational and translational components on all the morphed templates so that the mean shape could be determined and removed. The remaining zero mean data matrix served as a basis for the principal component analysis. To counterbalance higher point density in the facial regions, we used weighted PCA instead of ordinary PCA for the statistical modeling. The weights were assigned according to the surface area that each point encapsulated and computed using the area of each triangle of the surface model. We created one SSM for each class, ensuring that the models were independent from each other and did not contain influences from the other classes. We cut off the coefficient vectors after 95% of the normalized variance to remove noise and ensured only the most important components were included in the SSMs. The synthesis of the model instances could then be performed as

(1) $$\;s=\bar{\;s}+\;V{\;\Lambda }^{1/2}\;\alpha ,$$

with $\bar{s}$ denoting the mean shape, V the principal components, Λ the sample covariance matrix, and α the shape coefficient vector. We created 1000 random shapes of each class using a Gaussian distribution of the shape coefficient vector and created craniosynostosis distance maps for each sample.

#### 2.3.2. Image-based principal component analysis

We used ordinary PCA as another modality to generate 2D images. While the SSM also made use of PCA in the 3D domain, image-based PCA operated directly on the 2D images. This was a computationally inexpensive and less sophisticated alternative to both GANs and SSMs since neither extensive model training and hyperparameter tuning nor 3D morphing and correspondence establishment were required. We employed ordinary PCA for each of the four classes separately and we again created 1,000 samples for each class. Since SSM is related to PCA, the image synthesis could be performed as

(2) $$\;i=\bar{\;i}+\;V{\;\Lambda }^{1/2}\;\alpha ,$$

with $\bar{i}$ denoting the mean image in vectorized shape, V again the principal components, Λ the sample covariance matrix, and α the coefficient vector of the principal components. We again drew 1,000 random vectors from a Gaussian distribution and transformed them back into 2D image-shape.

#### 2.3.3. Generative adversarial network

**Figure 6:**
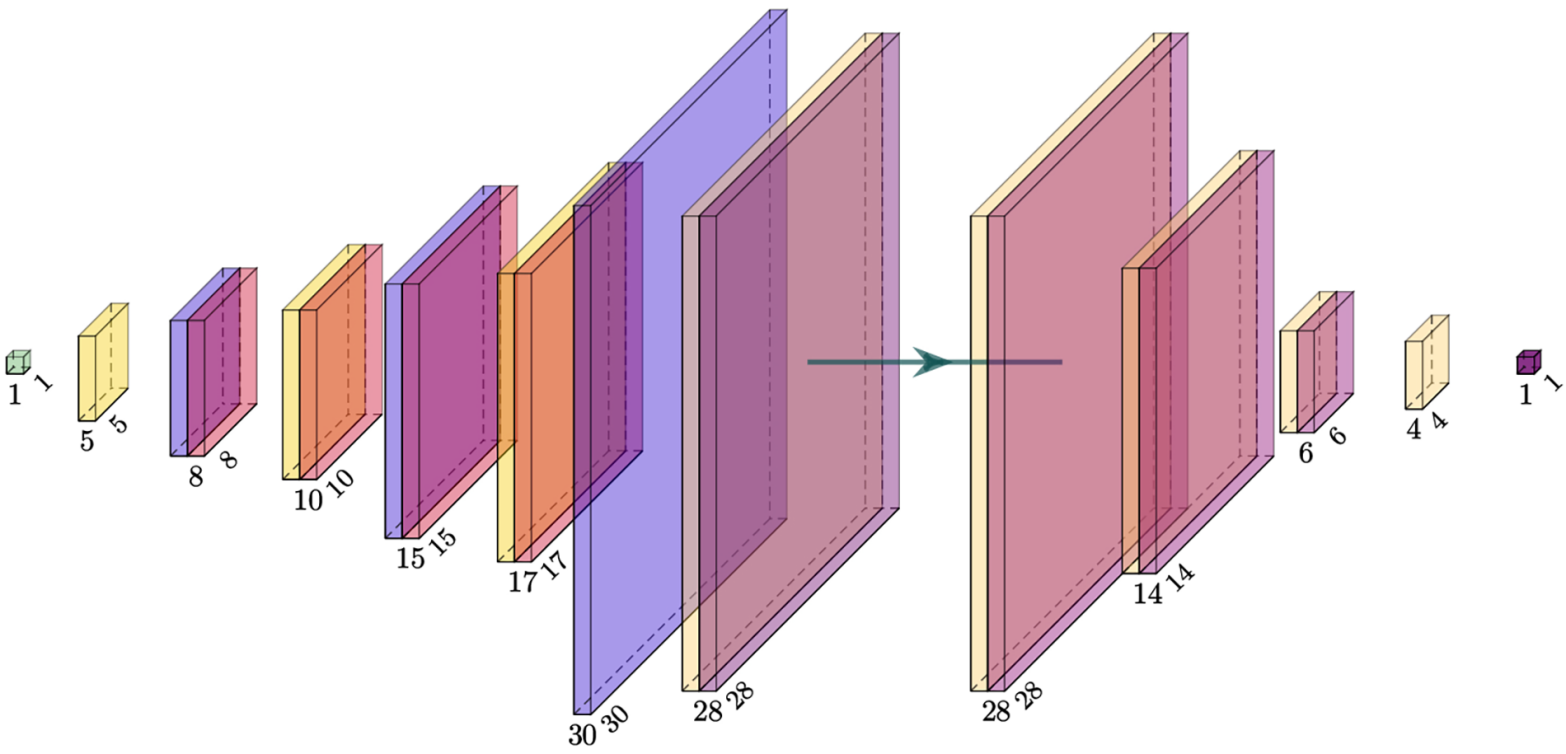
Visualization of the intermediate image sizes from the used GAN model. Left: generator; right: critic (discriminator). The filter kernel sizes are described in Appendix 1 in the Supplementary Material.

The GAN combined multiple suggestions from different GAN designs and was designed as a conditional (28) deep convolutional (29) Wasserstein (30) GAN with gradient penalty (31) (cDC-WGAN-GP). The design in terms of the intermediate image sizes is visualized in Figure 6. For the full design including all layers, please see Appendix 1 in the Supplementary Material.

**Figure 7:**
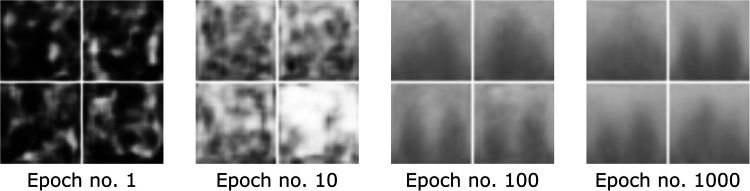
Image development of the GAN generator during different stages of training visualized as a 2×2 grid.

We opted for a design including a mixture between transposed, interpolation, and normal convolutional filter kernels, which prevented checkerboard artifacts and large patches. The combination of interpolation layers and transposed convolutional layers lead to better images than each of the approaches alone (see also in Appendix 1 in the Supplementary Material, Figure S1) present in our previous approach (32). The conditioning of the GAN was implemented as an embedding vector controlling the image label that we wished to synthesize. We trained the GAN for 1,000 epochs using the Wasserstein distance (30), which is considered to stabilize training (33). Instead of the originally proposed weight clipping, we used a gradient penalty (31) of λ=1. We used 10 critic iterations before updating the generator and a learning rate of $\alpha =3\times 10^{-5}$ for both networks. The loss L can be described as follows (31):

(3) $$L=E_{\tilde{x}∼E_{D}}D(\tilde{x}\;|\;y)-E_{x∼E_{G}}D(x\;|\;y)+\lambda (\|\nabla_{\hat{x}}D(\hat{x}){\|}_{2}-1{)}^{2}$$

with $\tilde{x}$ denoting the generator samples G(z|y) and $\hat{x}=\epsilon x+(1-\epsilon )\tilde{x}$ with ϵ denoting a uniformly distributed random variable between 0 and 1 (31). Exemplary synthetic images created by the GAN during different stages of training are depicted in Figure 7.

### 2.4. Image assessment

To assess image similarity, structural similarity index measure (SSIM) is one of the most popular metrics (34). Our goal was a metric to assess image similarity from the synthetic images to its clinical images from the same class. We adjusted the metric and computed the SSIM for each synthetic image to all the clinical samples from the same class and selected the maximum value for each synthetic image and defined it as its structural similarity index measure to closest clinical sample (SSIM_cc_). This way, the SSIM_cc_ is 1 if it matches any of the clinical samples from the same class and ≈ 0 if it is very dissimilar to any of them:

(4) $${\mathrm{SSIM}}_{\mathrm{cc},i}=\max_{\forall n\in N}\mathrm{SSIM}(p_{i,\mathrm{synthetic}},p_{n,\mathrm{clinical}})$$

with i denoting a synthetic image and n denoting the clinical index of the same class among the N clinical samples.

### 2.5. CNN training

Resnet18 was used as a classifier since it showed the best performance on this type of distance maps (17). We used pytorch’s (35) publicly available, pre-trained Resnet18 model and fine-tuned the weights during training. During training, all images were reshaped to a size of 224×224 to match the input size of Resnet18. We performed a different run of CNN training on all seven combinations of the synthetic images. Since we used fourfold cross-validation, this yielded four results for one of the synthetic image combinations.

Since we aimed to compare different synthetic data sources, the CNNs were trained on the synthetic training set and the best-performing network was chosen according to the maximum F1 score on the validation set. The validation set was the 2D image representation of the full “model data.” The test set was never touched during training and only evaluated in a final run after training and was composed of the 2D image representation of the “evaluation data.”

**Figure 8:**
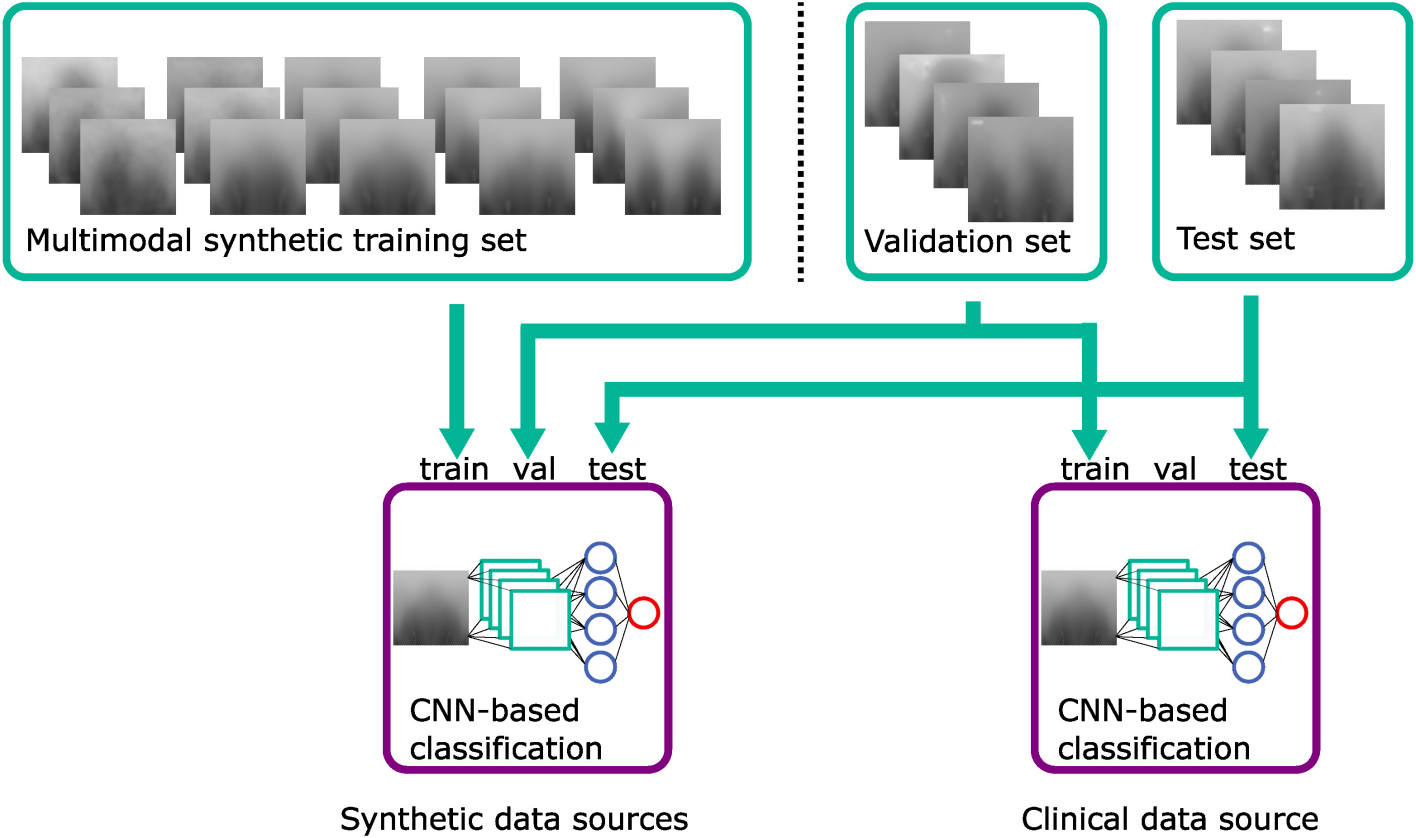
Classification training using the synthetic training set, the validation set, and the test set. The CNN classifier using clinical data uses the validation set as a training set. Green: datasets; blue: violet: classification models.

To evaluate the synthetically trained models against a clinically trained model, we additionally employed one CNN trained on the 2D images of the full “model data,” which had been used as the validation set for the synthetically trained CNNs and also tested it on the 2D images of the “evaluation data” to have the same test set as for the other CNNs. This is visualized in Figure 8.

**Figure 9:**
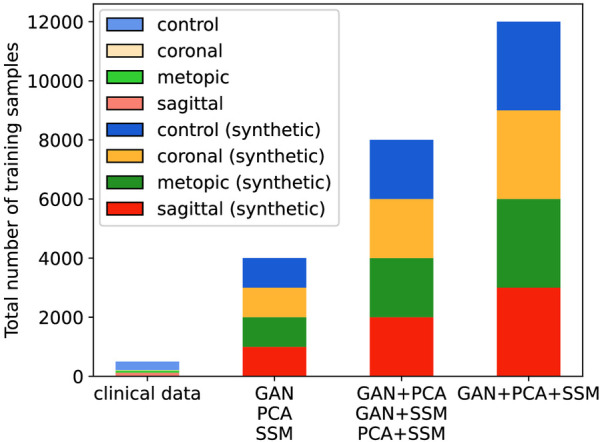
Number of training samples in each classification scenario. The clinical scenario has 372 samples during each cross-validation fold, while all synthetic scenarios have 4,000, 8,000, or 12,000 samples.

When multiple data sources were used, the models had a different number of training samples (see Figure 9), and all synthetically trained models were trained for 50 epochs. Convergence was achieved usually already during the first 10 epochs, indicating that there was sufficient training material for each model. We used the Adam optimizer, cross entropy loss, a batch size of 32 with a learning rate of $1\times 10^{-4}$, and a weight decay of 0.63 after each five epochs.

We used the following types of data augmentation during training: Adding random pixel noise (with σ=1/255), adding a random intensity (with σ=5/255) across all pixels, horizontal flipping, and shifting images left or right (with σ=12.44pixels). All those types of data augmentation corresponded to real-world patient and scanning modifications: Pixel noise corresponded to scanning and resolution errors, adding a constant intensity was equal to a re-scaling of the patient’s head, horizontal flipping corresponded to the patient as if they were mirrored in real life, and shifting the image horizontally modeled an alignment error in which the patient effectively turns their head $20^{\circ }$ left or right during recording.

All the clinical 2D images, the GAN, and the statistical models were made publicly available.^1^ We included a script to create synthetic samples for all three image modalities to allow users to create a large number of samples. The synthetic and clinical samples of this study are available on Zenodo (36).

## 3. Results

### 3.1. Image evaluation

**Figure 10:**
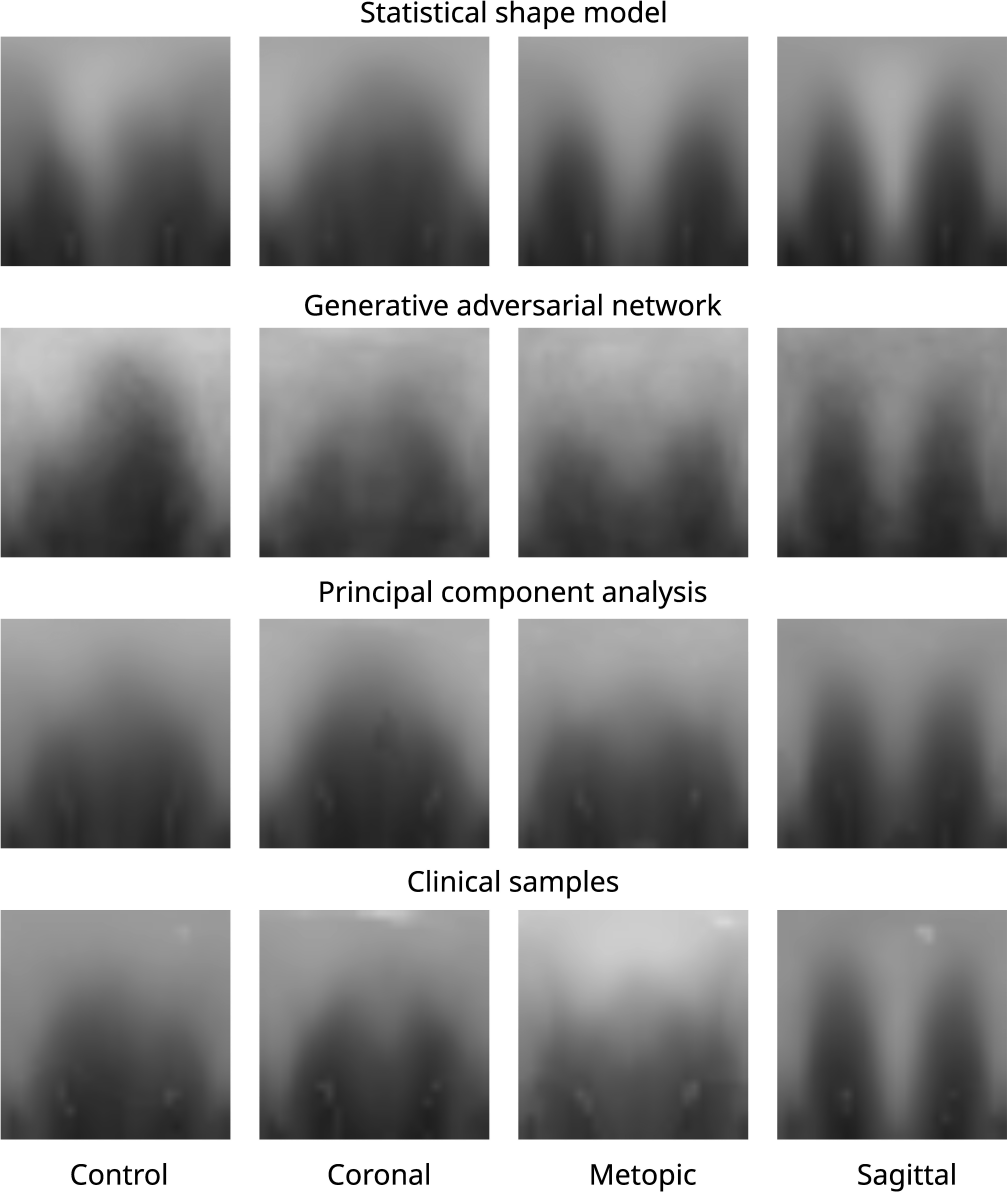
Images of all three data modalities and clinical samples. The image modalities from top to bottom: SSM, GAN, PCA, and clinical. The four classes from left to right: control, coronal, metopic, and sagittal.

Figure 10 shows images of each of the different data synthesis types compared with clinical images. From a qualitative, visual examination, the synthetic images had similar color gradients, shapes, and intensities as the clinical images. GAN images appeared slightly noisier than the other images and did not show the left and right ear visible in the other images.

**Figure 11:**
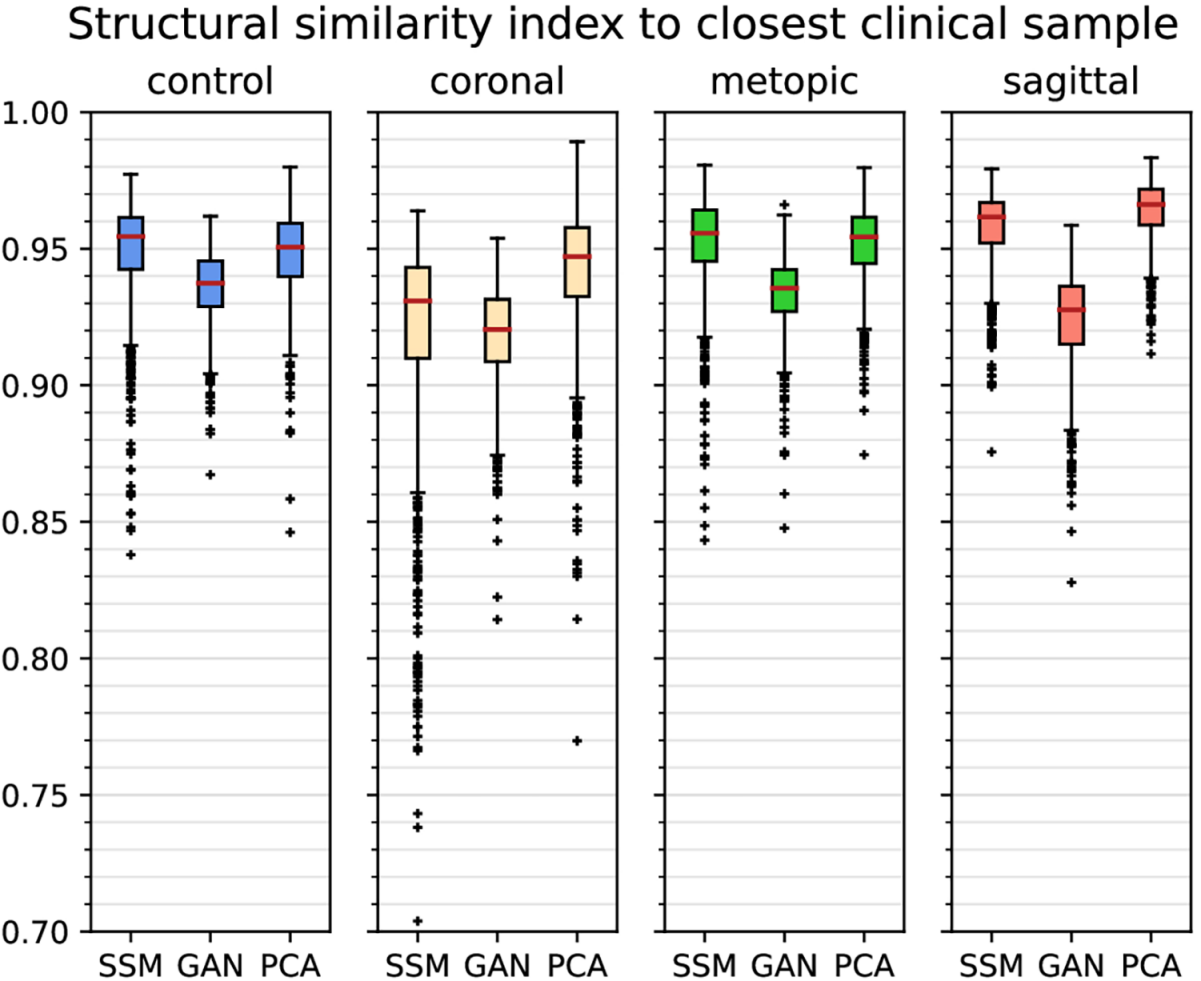
Boxplots of ${\mathrm{SSIM}}_{\mathrm{cc}}$ (structural similarity index measure to the closest clinical sample) of each class and each of the synthetic data generators. Outliers are marked with crosses, and the median is shown with a red bar.

From the quantitative comparison (see Figure 11), the GAN images showed the least similarity to the clinical images among all classes. The images of the coronal class were the least similar images among all classes. Overall, median SSIM_cc_ was above 0.92 for all classes and all synthetic modalities.

### 3.2. Classification results

All comparison presented here were carried out on the untouched test set and are displayed in Table 1. When considering only synthetic training images, the highest mean F1 score was obtained by the combination of the GAN and the SSM (0.929), which is slightly higher than the combination of all three synthetic image modalities (0.928) but slightly lower than using clinical images (0.931). The SSM scored the highest mean F1 score on the single image sources (0.823), while including a second image sources always led to mean F1 scores higher than 0.9. In contrast, the single synthetic data sources led to mean F1 scores from 0.657 (GAN) to 0.823 (SSM).

**Table 1:** CNN-classification comparison on the test set trained on different synthetic data sources.

Data source	Accuracy	F1-score
GAN	0.738±0.146	0.657±0.066
PCA	0.742±0.097	0.687±0.100
SSM	0.885±0.030	0.823±0.031
GAN-PCA	0.944±0.017	0.901±0.042
GAN-SSM	0.956±0.009	**0.929 ± 0.027**
PCA-SSM	0.942±0.015	0.908±0.034
GAN-PCA-SSM	**0.960 ± 0.014**	0.928±0.022
Clinical	0.968±0.013	0.931±0.033

The mean metric from fourfold cross-validation and the standard deviation are shown. Bold values represent best results overall, and bold italic values represent best result on the synthetic data source.

The mean accuracy of the three synthetic image sources scored highest among the synthetically trained classification (0.960), while still being slightly inferior to using clinical images (0.968). Mean accuracy was 0.944 or higher when using two synthetic image sources and in the range of 0.728 (GAN) to 0.885 (SSM) for a single synthetic image source. However, mean accuracy values are a less reliable metric compared to the F1 score due to the high dataset imbalance.

## 4. Discussion

Without being trained on a single clinical sample, the CNN trained from the combination of a PCA, an SSM, and a GAN was able to correctly classify 96% of the images. Classification performance on the synthetic images proved to be very close compared to training on the clinical images using the SSM and the GAN (and optionally also PCA). This suggests that certain combinations of synthetic data might be indeed sufficient for a classification algorithm to distinguish between types of craniosynostosis. Compared with classification results from other works, the accuracy of the purely synthetic data–based classification (96.8%) performed in a similar range to other approaches on clinical data such as 90.1% (37), 95.7% (13), 97.8% (16), 98.4% (17), and 99.5% (15). It has to be noted that all those experiments were computed on different datasets and a quantitative comparison does not indicate a better model. However, it does show that the classification approach on purely synthetic training data achieves performances in a similar range to models from the literature trained on clinical data.

The number of training samples also increased with the combination of synthetic image sources; both the number of training samples and the type of synthetic images might have played a role for an increased classification performance using a GAN, PCA, and SSM. However, the best F1 score on synthetic images was achieved by the combination of only GAN and SSM, so the increased number of samples was likely not the primary influence on the classification accuracy. The SSM appeared to be the data source contributing the most to the improvement of the classifier: Not only did it score highest among the unique data sources, but it was also present in the highest scoring classification approaches according to its F1 score. As the SSM models 3D shapes, the 2D distance maps derived from the SSM are always valid 3D samples, while PCA and the GAN could, in theory, create 2D images, which do not correspond to a valid 3D shape. In contrast, the GAN-based classifiers only showed a good classification performance when combined with a different data modality and its synthesized images seemed to show less pronounced visual features than the other two modalities. Possible reasons include that the SSIM_cc_ was lowest for the GAN, and since one conditional GAN synthesized images for all pathologies, the images might still contain features that are derived from images from other classes. The PCA images were neither required nor detrimental for a good classification performance.

By itself, none of the synthetic data sources was an adequate replacement for clinical data. However, a combination of different data modalities seemed to be the key element for achieving a good classification performance. Both SSM and PCA model the data according to a Gaussian distribution, while the GAN uses an unrestricted distribution model. The different properties of modeling the underlying statistical distribution of a Gaussian distribution (SSMs and PCA), on the one hand, and without an assumed distribution (GAN), on the other hand, might have led to a compensation of their respective disadvantages increasing the overall performance for the combinations. One limitation of this study is the small dataset. As the clinical classification uses the same dataset for training and validation, this might make it prone to overfitting. However, the resulting classification metrics achieved in this study were similar to a classification study on clinical data alone (17) (accuracy: 0.984 and F1 score: 0.964), which suggests that overfitting has not been an issue. In addition, all clinical data were acquired in the same clinical center, which might make the classification models less robust when compared to data acquired in other hospitals. Since the trained models are publicly available, they can be tested by other groups on their own data.

## 5. Conclusion

We showed that it is possible to train a classifier for different types of craniosynostosis based solely on artificial data synthesized by an SSM, a PCA, and a GAN. Without having seen any clinical samples, a CNN was able to classify four types of head deformities with an F1 score of 0.929 and performed comparable to a classifier trained on clinical data. The key component in achieving good classification results was using multiple but different data generation models. Overall, the SSM was the data source contributing most to the classification performance. For the GAN, using a small image size and alternating between transposed convolutions and interpolations were identified as key elements for suitable image generation. Datasets and generators were made publicly available along with this work. We showed that clinical data are not required for the classification of craniosynostosis paving the way into the cost-effective usage of synthetic data for automated diagnosis systems.

## Data availability statement

The original contributions presented in the study are included in the article/Supplementary Material, further inquiries can be directed to the corresponding author.

## Ethics statement

The studies involving humans were approved by the Ethics Committee Medical Faculty of the University of Heidelberg (Ethics number S-237/2009). Written informed consent to participate in this study was provided by the participants’ legal guardian/next of kin. The studies were conducted in accordance with the local legislation and institutional requirements. Written informed consent for participation in this study was provided by the participants’ legal guardians/next of kin.

## Footnotes

^1^ https://github.com/KIT-IBT/craniosource-gan-pca-ssm

## References

[1] French LR Jackson IT Melton LJ. A population-based study of craniosynostosis. J Clin Epidemiol. (1990) 43(1):69–73. 10.1016/0895-4356(90)90058-W 2319283

[2] Persing JA Jane JA Shaffrey M. Virchow, the pathogenesis of craniosynostosis: a translation of his original work. Plast Reconstr Surg. (1989) 83(4):738–42. 10.1097/00006534-198904000-00025 2648432

[3] Coussens AK Wilkinson CR Hughes IP Morris CP van Daal A Anderson PJ, et al. Unravelling the molecular control of calvarial suture fusion in children with craniosynostosis. BMC Genomics. (2007) 8(1):458. 10.1186/1471-2164-8-458 18076769 PMC2222648

[4] Boulet SL Rasmussen SA Honein MA. A population-based study of craniosynostosis in metropolitan Atlanta, 1989–2003. Am J Med Genet A. (2008) 146(8):984–91. 10.1002/ajmg.a.32208 18344207

[5] Renier D Sainte-Rose C Marchac D Hirsch J-F. Intracranial pressure in craniostenosis. J Neurosurg. (1982) 57(3):370–7. 10.3171/jns.1982.57.3.0370 7097333

[6] Kapp-Simon KA Speltz ML Cunningham ML Patel PK Tomita T. Neurodevelopment of children with single suture craniosynostosis: a review. Childs Nerv Syst. (2007) 23(3):269–81. 10.1007/s00381-006-0251-z 17186250

[7] Fearon JA Ruotolo RA Kolar JC. Single sutural craniosynostoses: surgical outcomes and long-term growth. Plast Reconstr Surg. (2009) 123(2):635–42. 10.1097/PRS.0b013e318195661a 19182624

[8] Saarikko A Mellanen E Kuusela L Leikola J Karppinen A Autti T, et al. Comparison of black bone MRI and 3D-CT in the preoperative evaluation of patients with craniosynostosis. J Plast Reconstr Aesthet Surg. (2020) 73(4):723–31. 10.1016/j.bjps.2019.11.006 31917189

[9] Barbero-García I Lerma JL Mora-Navarro G. Fully automatic smartphone-based photogrammetric 3D modelling of infant’s heads for cranial deformation analysis. ISPRS J Photogramm Remote Sens. (2020) 166:268–77. 10.1016/j.isprsjprs.2020.06.013

[10] Schaufelberger M Schaufelberger M Kühle RP Wachter A Weichel F Hagen N, et al. A statistical shape model of craniosynostosis patients and 100 model instances of each pathology. Zenodo. (2021). 10.5281/zenodo.10167123

[11] Nagel C Schaufelberger M Dössel O Loewe A. A bi-atrial statistical shape model as a basis to classify left atrial enlargement from simulated, clinical 12-lead ECGs. *Statistical atlases and computational models of the heart. Multi-disease, multi-view, and multi-center right ventricular segmentation in cardiac MRI challenge*. Vol. 13131. Cham: Springer International Publishing (2022). p. 38–47.

[12] Sánchez J Luongo G Nothstein M Unger LA Saiz J Trenor B, et al. Using machine learning to characterize atrial fibrotic substrate from intracardiac signals with a hybrid in silico and in vivo dataset. Front Physiol. (2021) 12:699291. 10.3389/fphys.2021.699291 34290623 PMC8287829

[13] Mendoza CS Safdar N Okada K Myers E Rogers GF Linguraru MG. Personalized assessment of craniosynostosis via statistical shape modeling. Med Image Anal. (2014) 18(4):635–46. 10.1016/j.media.2014.02.008 24713202

[14] Tabatabaei SAH Fischer P Wattendorf S Sabouripour F Howaldt H-P Wilbrand M, et al. Automatic detection and monitoring of abnormal skull shape in children with deformational plagiocephaly using deep learning. Sci Rep. (2021) 11(1):17970. 10.1038/s41598-021-96821-7 34504140 PMC8429738

[15] de Jong G Bijlsma E Meulstee J Wennen M van Lindert E Maal T, et al. Combining deep learning with 3D stereophotogrammetry for craniosynostosis diagnosis. Sci Rep. (2020) 10(1):15346. 10.1038/s41598-020-72143-y 32948813 PMC7501225

[16] Schaufelberger M Kühle R Wachter A Weichel F Hagen N Ringwald F, et al. A radiation-free classification pipeline for craniosynostosis using statistical shape modeling. Diagnostics. (2022) 12(7):1516. 10.3390/diagnostics12071516 35885422 PMC9323148

[17] Schaufelberger M Kaiser C Kühle R Wachter A Weichel F Hagen N, et al. 3D-2D distance maps conversion enhances classification of craniosynostosis. IEEE Trans Biomed Eng. (2023) 70:1–10. 10.1109/TBME.2023.3278030 37204949

[18] Meulstee JW Verhamme LM Borstlap WA Van der Heijden F De Jong GA Xi T, et al. A new method for three-dimensional evaluation of the cranial shape and the automatic identification of craniosynostosis using 3D stereophotogrammetry. Int J Oral Maxillofac Surg. (2017) 46(7):819–26. 10.1016/j.ijom.2017.03.017 28392059

[19] Rodriguez-Florez N Bruse JL Borghi A Vercruysse H Ong J James G, et al. Statistical shape modelling to aid surgical planning: associations between surgical parameters and head shapes following spring-assisted cranioplasty. Int J Comput Assist Radiol Surg. (2017) 12(10):1739–49. 10.1007/s11548-017-1614-5 28550406 PMC5608871

[20] Heutinck P Knoops P Florez NR Biffi B Breakey W James G, et al. Statistical shape modelling for the analysis of head shape variations. J Cranio-Maxillofac Surg. (2021) 49(6):449–55. 10.1016/j.jcms.2021.02.020 33712336

[21] Goodfellow I Pouget-Abadie J Mirza M Xu B Warde-Farley D Ozair S Courville A Bengio Y. Generative adversarial networks. Commun. ACM. (2020) 63(11):139–44.

[22] Pinetz T Ruisz J Soukup D. Actual impact of GAN augmentation on CNN classification performance. *Proceedings of the 8th International Conference on Pattern Recognition Applications and Methods; 2019 Feb 19–21; Prague*. Prague, Czech Republic: SCITEPRESS - Science and Technology Publications (2019). p. 15–23.

[23] Cignoni P Callieri M Corsini M Dellepiane M Ganovelli F Ranzuglia G. MeshLab: an open-source mesh processing tool. *Eurographics Italian Chapter Conference; 2008 Jul 2; Salerno, Italy*. Salerno, Italy: The Eurographics Association (2008). 8 pages.

[24] Pietroni N Tarini M Cignoni P. Almost isometric mesh parameterization through abstract domains. IEEE Trans Vis Comput Graph. (2010) 16(4):621–35. 10.1109/TVCG.2009.96 20467060

[25] Dai H Pears N Smith W Duncan C. A 3D morphable model of craniofacial shape and texture variation. *2017 IEEE International Conference on Computer Vision (ICCV)*; 2017 Oct; Venice, Italy. Danvers, MA: IEEE (2017). p. 3104–12.

[26] Dai H Pears N Smith W. Augmenting a 3D morphable model of the human head with high resolution ears. Pattern Recognit Lett. (2019) 128:378–84. 10.1016/j.patrec.2019.09.026

[27] Dai H Pears N Smith W Duncan C. Statistical modeling of craniofacial shape and texture. Int J Comput Vis. (2020) 128(2):547–71. 10.1007/s11263-019-01260-7

[28] Mirza M Osindero S. Conditional generative adversarial nets. arXiv Preprint. (2014). arXiv:1411.1784.

[29] Radford A Metz L Chintala S. Unsupervised representation learning with deep convolutional generative adversarial networks. arXiv Preprint. (2015). arXiv:1511.06434.

[30] Arjovsky M Chintala S Bottou L. Wasserstein GAN. arXiv Preprint. (2017). arXiv:1701.07875

[31] Gulrajani I Ahmed F Arjovsky M Dumoulin V Courville AC. Improved training of wasserstein gans. arXiv Preprint. (2017). arXiv:1704.00028.

[32] Kaiser C Schaufelberger M Kühle RP Wachter A Weichel F Hagen N, et al. Generative-adversarial-network-based data augmentation for the classification of craniosynostosis. Curr Dir Biomed Eng. (2022) 8(2):17–20. 10.1515/cdbme-2022-1005

[33] Arjovsky M Bottou L. Towards principled methods for training generative adversarial networks. arXiv Preprint. (2017). arXiv:1701.04862.

[34] Wang Z Bovik AC Sheikh HR Simoncelli EP. Image quality assessment: from error visibility to structural similarity. IEEE Trans Image Process. (2004) 13(4):600–12. 10.1109/TIP.2003.819861 15376593

[35] Paszke A Gross S Massa F Lerer A Bradbury J Chanan G, et al. Pytorch: an imperative style, high-performance deep learning library. In: Wallach HM, Larochelle H, Beygelzimer A, d'Alché-Buc F, Fox EB, editor. *Advances in neural information processing systems 32*; 2019 Dec 8; Vancouver, CB, Canada. New York: Curran Associates, Inc. (2019). p. 8024–35.

[36] Schaufelberger M Kühle R Wachter A Weichel F Hagen N Ringwald F, et al. GAN, PCA, and statistical shape models for the creation of synthetic craniosynostosis distance maps. Zenodo (2023). 10.5281/zenodo.8117499

[37] Agarwal S Hallac RR Daescu O Kane A. Classification of craniosynostosis images by vigilant feature extraction. In: *Advances in computer vision and computational biology*. Cham: Springer International Publishing (2021). p. 293–306.

