# Supplementary material for: Impact of data synthesis strategies for the classification of craniosynostosis
Source: Front Med Technol. 2023 Dec 13;5:1254690. doi: 10.3389/fmedt.2023.1254690 (PMC10773901; doi:10.3389/fmedt.2023.1254690)
Supplement: Supplementary file 1 [file Datasheet1.pdf]

1 GENERATIVE ADVERSARIAL NETWORK STRUCTURE

This is the GAN structure of generator and discriminator employed for the creation of the synthetic data as an output of according to models' `str__` method called via `print(model)`.

```
Generator28(
  (embed): Embedding(4, 100)
  (gen): Sequential(
    (0): Sequential(
      (0): ConvTranspose2d(200, 256, kernel_size=(5, 5), stride=(1, 1),
              bias=False)
      (1): BatchNorm2d(256, eps=1e-05, momentum=0.1, affine=True,
              track_running_stats=True)
      (2): ReLU(inplace=True)
    )
    (1): Sequential(
      (0): Interpolate(size=(8, 8),bilinear,align_corners=True)
      (1): BatchNorm2d(256, eps=1e-05, momentum=0.1, affine=True,
              track_running_stats=True)
      (2): ReLU(inplace=True)
    )
    (2): Sequential(
      (0): Conv2d(256, 128, kernel_size=(3, 3), stride=(1, 1), padding=(1, 1),
              bias=False)
      (1): BatchNorm2d(128, eps=1e-05, momentum=0.1, affine=True,
              track_running_stats=True)
      (2): ReLU(inplace=True)
    )
    (3): Sequential(
      (0): Interpolate(size=(15, 15),bilinear,align_corners=True)
      (1): BatchNorm2d(128, eps=1e-05, momentum=0.1, affine=True,
              track_running_stats=True)
      (2): ReLU(inplace=True)
    )
    (4): Sequential(
      (0): ConvTranspose2d(128, 128, kernel_size=(3, 3), stride=(1, 1),
              bias=False)
      (1): BatchNorm2d(128, eps=1e-05, momentum=0.1, affine=True,
              track_running_stats=True)
      (2): ReLU(inplace=True)
    )
    (5): Sequential(
      (0): Interpolate(size=(30, 30),bilinear,align_corners=True)
      (1): BatchNorm2d(128, eps=1e-05, momentum=0.1, affine=True,
              track_running_stats=True)
      (2): ReLU(inplace=True)
    )
    (6): Conv2d(128, 1, kernel_size=(3, 3), stride=(1, 1),
              bias=False)
    (7): Tanh()
  )
)

Discriminator28(
```

```
  (net): Sequential(
    (0): Sequential(
      (0): Conv2d(2, 32, kernel_size=(4, 4), stride=(2, 2), padding=(1, 1),
              bias=False)
      (1): InstanceNorm2d(32, eps=1e-05, momentum=0.1, affine=True,
              track_running_stats=False)
      (2): LeakyReLU(negative_slope=0.2)
    )
    (1): Sequential(
      (0): Conv2d(32, 128, kernel_size=(4, 4), stride=(2, 2), padding=(1, 1),
              bias=False)
      (1): InstanceNorm2d(128, eps=1e-05, momentum=0.1, affine=True,
              track_running_stats=False)
      (2): LeakyReLU(negative_slope=0.2)
    )
    (2): Sequential(
      (0): Conv2d(128, 256, kernel_size=(5, 5), stride=(2, 2), padding=(1, 1),
              bias=False)
      (1): InstanceNorm2d(256, eps=1e-05, momentum=0.1, affine=True,
              track_running_stats=False)
      (2): LeakyReLU(negative_slope=0.2)
    )
    (3): Conv2d(256, 1, kernel_size=(3, 3), stride=(1, 1))
  )
  (embed): Embedding(4, 784)
)
```

2 FAILED GAN ATTEMPTS

Fig. 12 displays artifacts arising from poor training: First using only transposed convolutional layers (`ConvTranspose2d`), second using only up-scaling interpolation layers (`Interpolate`), and third from using large gradient penalties which prohibits training.

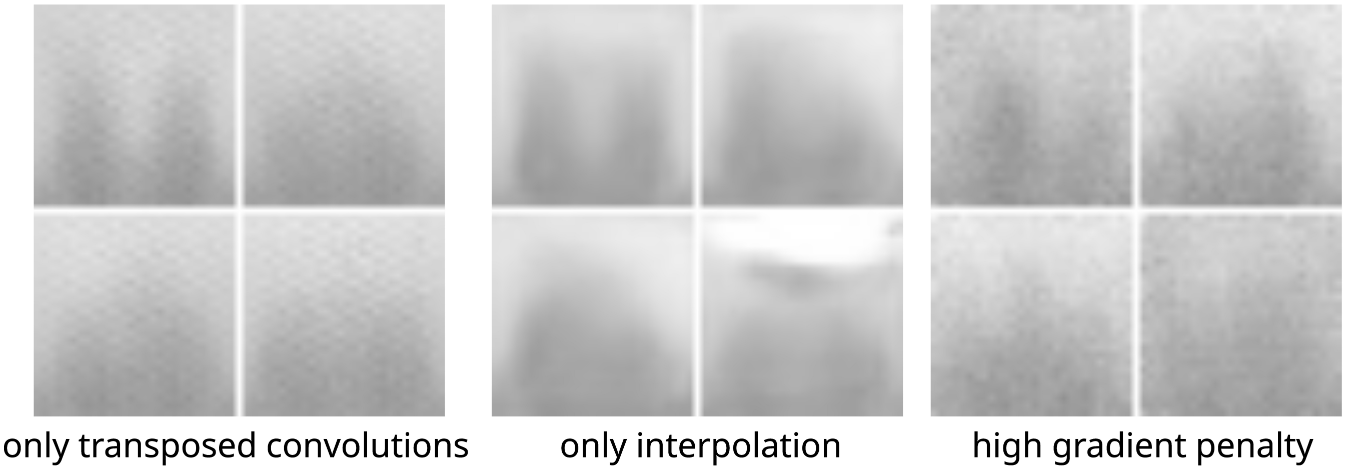

Figure 12. Failed GAN images arranged in a 2×2 grid with artifacts arising from poor network design or bad training conditions. From left to right: Deconvolution artifacts, interpolation artifacts, and noise artifacts.
